# Endothelin-receptor antagonists for aneurysmal subarachnoid hemorrhage: an updated meta-analysis of randomized controlled trials

**DOI:** 10.1186/cc11686

**Published:** 2012-10-18

**Authors:** Junpeng Ma, Siqing Huang, Lu Ma, Yi Liu, Hao Li, Chao You

**Affiliations:** 1 Department of Neurosurgery, West China Hospital, Sichuan University, 37 Guoxuexiang Street, Chengdu 610041, P.R. China

## Abstract

**Introduction:**

The previous meta-analysis on the use of endothelin-receptor antagonists (ETRAs) to treat aneurysmal subarachnoid hemorrhage (SAH) has become outdated due to recently published phase 3 clinical trials. An up-to-date meta-analysis is needed to provide the best available evidence for the efficacy of ETRAs for aneurysmal SAH.

**Methods:**

We performed a systematic review and meta-analysis of published randomized controlled trials that investigate efficacy of ETRAs in patients with aneurysmal SAH. Mortality, unfavorable outcome, delayed ischemic neurological deficit (DIND), delayed cerebral infarction (DCI), angiographic vasospasm and adverse events were analyzed. Meta-analysis was performed in terms of the risk ratio (RR) and 95% confidence interval (CI).

**Results:**

Five eligible studies were reviewed and analyzed, involving 2,595 patients. The pooled RRs of mortality and unfavorable outcome after SAH were 1.03 (95% CI = 0.77 to 1.36) and 1.07 (95% CI = 0.93 to 1.22), respectively. The pooled RRs were 0.87 (95% CI = 0.74 to 1.03) for DCI, 0.77 (95% CI = 0.66 to 0.90) for DIND, and 0.66 (95% CI = 0.57 to 0.77) for angiographic vasospasm. There were significant increases in lung complications (RR = 1.80, 95% CI = 1.55 to 2.09), hypotension (RR = 2.42, 95% CI = 1.78 to 3.29) and anemia (RR = 1.47, 95% CI = 1.19 to 1.83) in patients administered ETRAs.

**Conclusion:**

There is no evidence that ETRAs could benefit clinical outcome in patients with SAH. Owing to the increased adverse events, further clinical trials of ETRAs in SAH patients should be more carefully formulated and designed. The present results also suggest that DCI may be a better outcome measure than vasospasm and DIND in SAH clinical trials and observational studies.

## Introduction

Spontaneous subarachnoid hemorrhage (SAH) is the extravasation of blood into the subarachnoid spaces filled with cerebrospinal fluid [1]. SAH accounts for 2 to 5% of all new strokes, with an overall incidence of about 9 per 100,000 person-years [2]. The outcome of patients after SAH is generally poor: about one-half of patients died after SAH and many survivors have long-term cognitive and neurological impairment [3-5]. Vasospasm is one of the most serious complications after SAH, found in up to 70% of patients with SAH on angiography Vasospasm-associated clinical manifestation, delayed ischemic neurological deficit (DIND) and delayed cerebral infarction (DCI) are the leading causes of mortality and morbidity after SAH and are widely considered potential therapeutic targets [6,7]. Clinical trials testing medical treatments for prevention of vasospasm have been disappointing, with only hemodynamic therapy and calcium antagonists having a beneficial effect, but the efficacy of these treatments is suboptimal [8-11].

Recently, there has been increasing interest in the use of endothelin-receptor antagonists (ETRAs) to prevent endothelin-mediated cerebral vasospasm after aneurysmal SAH. Endothelin is one of the most potent endogenous vasoconstrictors, which is responsible for the clinical manifestation of cerebral vasospasm following SAH [12]. Both experimental and clinical data demonstrate increased cerebrospinal fluid and plasma levels of endothelin expression in the presence of vasospasm. There is also an increase of endothelin-receptor expression in cerebral arteries following SAH [13,14]. Previous clinical trials and meta-analysis indicated that ETRAs can prevent both radiographic vasospasm and DINDs, but did not demonstrate that they can improve clinical outcomes [15]. Owing to recently published phase 3 clinical trials, the previous meta-analysis on the use of ETRAs to treat aneurysmal SAH has become outdated [16]. To provide the best available evidence for the effect and safety of ETRAs for aneurysmal SAH, we conducted an up-to-date meta-analysis in the present article.

## Materials and methods

The present study was performed according to the PRISMA guidelines (Additional File 1). The review protocol has not been registered previously.

### Type of studies included

We included only published randomized controlled trials (RCTs) comparing ETRAs with no ETRAs or with placebo administration for patients with acute aneurysmal SAH.

### Types of outcome measures

The following outcomes were evaluated: mortality at the end of scheduled follow-up; unfavorable outcome (either death or dependency) at the end of scheduled follow-up (dependency assessed at least 1 month after SAH); DCI and DIND; angiographic vasospasm; and adverse events.

We defined dependency as being dependent on others for activities of daily living; for example, having Glasgow Outcome Scale score <4, Extended Glasgow Outcome Scale ≤4, modified Rankin Scale score graded 3 to 5, or Barthel Index 0 to 60 [17,18]. Angiographic vasospasm was defined as moderate (34 to 66%) or severe (67 to 100%) arterial narrowing on digital subtraction angiography determined by neuroradiologists [19]. DIND was defined by a delayed decrease of consciousness by at least two Glasgow Coma Scale levels and/or an increase ≥2 points on the abbreviated National Institutes of Health Stroke Scale and/or a new focal neurological deficit [16]. DCI was defined as confirmed new hypodensities on the computed tomography (CT) scan that were only attributable to cerebral vasospasm and delayed cerebral ischemia [20].

### Search strategy

We performed a systematical search of the Cochrane Central Register of Controlled Trials, PubMed, MEDLINE, EMBASE and Science Citation Index Expanded (SCI-EXPANDED) from 1980 to July 2012. The details of full electronic search strategies are presented in Additional File 2. The reference lists of all relevant papers and literature reviews were checked. In addition, we contacted researchers and study authors for further information where possible.

### Study selection and data extraction

Three review authors (JM, HL and SH) independently screened the titles, abstracts and keywords of citations obtained from the searches of the electronic databases and excluded studies that were clearly irrelevant. We obtained the full text of the remaining studies and the same three review authors independently assessed which trials met the predefined inclusion criteria. Disagreements were resolved by consensus between investigators. The following data of included studies were extracted independently by the same three authors: first author, year of publication, journal, study center, study population characteristics (inclusion and exclusion criteria, age, gender, similarity of groups at baseline, severity of SAH), sample size, interventions for ETRAs (time of initiated administration, type, dose, duration, route of administration), and treatment of aneurysm (clip or coil). The methodological quality of each trial was evaluated using the Jadad scale [21] and the risk of bias assessment tool in the *Cochrane Handbook for Systematic Reviews of Interventions *[22]. The criteria included randomization, allocation concealment, blinding, and an explanation of withdrawal or loss to follow-up. Clinical trials with Jadad scores ≥3 were considered to have lower bias risks. Any discrepancies were resolved by consensus between investigators.

### Statistical analysis and assessment for bias

Meta-analysis was performed to calculate the risk ratio (RR) and 95% confidence interval (CI) via a fixed-effect model if there is no evidence of statistical heterogeneity. The random-effects model was employed to pool studies when statistical heterogeneity occurred. We intended to performed subgroup analysis according to the type and dose of ETRAs, as well as the grade of SAH patients (high-grade patients vs. low-grade patients) and the treatment of intracranial aneurysm (clipped patients vs. coiled patients) if possible. We assessed and quantified statistical heterogeneity for each pooled summary estimate using Cochran's *Q *statistic and the *I*^2 ^statistic, respectively. Substantial heterogeneity will be considered to exist with *I*^2 ^>50% and chi-squared test *P*<0.1. All analyses were performed using the Review Manager Software, RevMan 5.1 (Version 5.1. Copenhagen: The Nordic Cochrane Centre, The Cochrane Collaboration, 2011).

## Results

### Trial selection and characteristics

**Figure 1:**
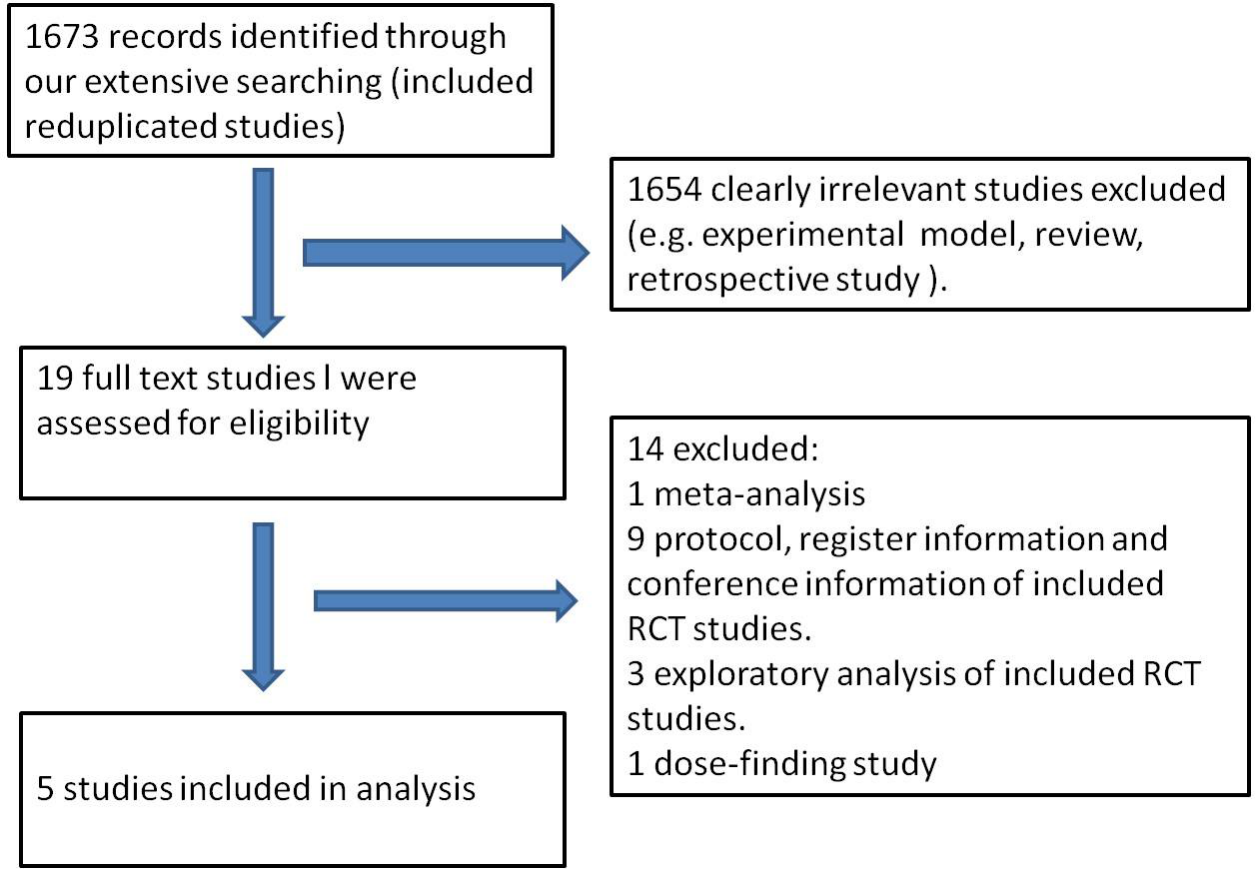
**Flow diagram for study selection**. RCT, randomized controlled trial.

The combined search strategy identified 1,673 citations (Figure 1). After title, abstract and full-text screening, five completed RCTs satisfied all inclusion criteria for final analysis and included a total of 2,595 patients [16,23-26]. Four trials, involving 2,175 patients, used the endothelin-A antagonist clazosentan compared with placebo. The other RCT enrolled 420 patients to receive either placebo or a mixed endothelin-A/B antagonist (TAK-044). Details of the included studies are presented in Table 1.

**Table 1:** Characteristics of the randomized controlled trials involving use of ETRAs in patients with SAH

Included study/year	Center/area	Participants (age/grade)	Number of participants (ETRA/placebo)	Treatment of aneurysm	ETRA (time of initiation, routine)	Dose (*n*)	Duration of treatment	Primary outcome	Secondary outcomes
Shaw and colleagues, 2000 [23]	Multicenter/Europe	>18 years old, WFNS 1 to 4 (79% grade 1 to 2)	420 (207/213)	75% clipped, 9% coiled	TAK-044 (within 96 hours, intravenously)	≤50 mg three times per day	10 days	DND within 3 months	DND within 10 days; DCI within 3 months; GOS at 3 months; adverse events
Vajkoczy and colleagues, 2005[24]	Multicenter/Germany	18 to 65 years old, HH 3 to 4	29 (13/16)	100% clipped	Clazosentan (within 48 hours, intravenously)	0.2 mg/kg/hour	14 days	Incidence and severity of angiographic vasospasm on day 8 post SAH	DCI at 14 days; adverse events
CONSCIOUS-1, 2008 [25]	Multicenter/North America and Europe	18 to 70 years old, WFNS 1 to 4 (74% grade 1 to 2)	409 (313/96)	45% clipped	Clazosentan (within 56 hours, intravenously)	1 mg/hour (108), 5 mg/hour (111), 15 mg/hour (98)	14 days	Moderate or severe vasospasm within 14 days	Morbidity and mortality at 6 and 12 weeks; DIND at 14 days; DCI at 6 weeks; rescue therapy within 14 days; adverse events
CONSCIOUS-2, 2011 [16]	Multicenter/North America and Europe	18 to 75 years old, WFNS 1 to 4 (78% grade 1 to 2)	1,147 (764/383)	100% clipped	Clazosentan (within 56 hours, intravenously)	5 mg/hour	14 days	Mortality and vasospasm-related morbidity within 6 weeks post SAH	Dichotomized GOSE score at 12 weeks; DCI, DIND, rescue therapy at 6 weeks; adverse events
CONSCIOUS-3, 2012 [26]	Multicenter/North America and Europe	18 to 75 years old, WFNS 1 to 4 (80% grade 1 to 2)	571 (382/189)	100% coiling	Clazosentan (within 56 hours, intravenously)	5 mg/hour (194), 15 mg/hour (188)	14 days	Mortality and vasospasm-related morbidity within 6 weeks post SAH	Dichotomized GOSE score at 12 weeks; DCI, DIND, rescue therapy at 6 weeks; death at 12 weeks; adverse events

DCI, delayed intracerebral infarction; DIND, delayed ischemic neurological deficit; DND, delayed neurological deterioration; ETRA, endothelin-receptor antagonist; GOS, Glasgow Outcome Scale; GOSE, Extended Glasgow Outcome Scale; HH, Hunt-Hess grade; SAH, subarachnoid hemorrhage; WFNS, World Federation of Neurological Surgeons grade.

### Assessment of trial quality

Five eligible studies were assessed for risks of bias using both the *Cochrane Handbook for Systematic Reviews of Interventions *and the Jadad scale. All these trials were found to have lower bias risks (Jadad score >3). Details of our assessment of the risk of bias in the included studies are presented in Table 2. We intended to access publication bias using a funnel plot and linear regression test, but there were too few included studies to enable meaningful analysis.

**Table 2:** Quality indicators and assessment of risk of bias in the included randomized controlled trials

Quality indicators/studies	**Shaw and colleagues **[23]	**Vajkoczy and colleagues **[24]	**CONSCIOUS-1 **[25]	**CONSCIOUS-2 **[16]	**CONSCIOUS-3 **[26]
Randomized controlled study	Yes	Yes	Yes	Yes	Yes
Appropriate random sequence generation	Yes	Unclear	Unclear	Yes	Yes
Allocation concealment	Unclear	Unclear	Unclear	Yes	Yes
Blinding of participants and personnel	Yes	Yes	Yes	Yes	Yes
Blinding of outcome assessment	Unclear	Yes	Yes	Yes	Yes
Explanation for withdrawals and dropouts	No	Yes	Yes	Yes	Yes
Jadad scale	4	4	4	5	5

### Effects of interventions

#### Mortality and unfavorable outcome

**Figure 2:**
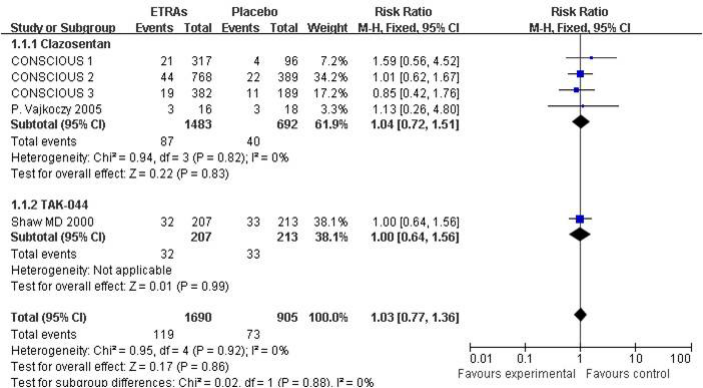
**Risk ratio for mortality at the end of follow-up**. Comparison between endothelin-receptor antagonists (ETRAs) and placebo in patients with aneurysmal subarachnoid hemorrhage and subgroup analysis according to type of ETRA. CI, confidence interval; df, degrees of freedom; M-H, Mantel-Haenszel.

**Figure 3:**
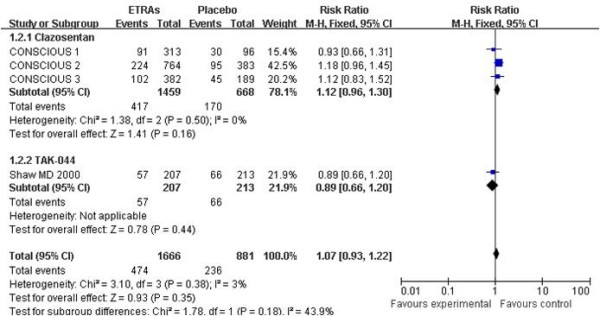
**Risk ratio for unfavorable outcome at the end of follow-up**. Comparison between endothelin-receptor antagonists (ETRAs) and placebo in patients with aneurysmal subarachnoid hemorrhage and subgroup analysis according to type of ETRA. CI, confidence interval; df, degrees of freedom; M-H, Mantel-Haenszel.

Five studies with 2,595 patients were available for analysis of mortality and four studies with 2,547 patients were available for analysis of unfavorable outcome (either death or dependency). The pooled RR for mortality at the end of scheduled follow-up was 1.03 (95% CI = 0.77 to 1.36). Heterogeneity: χ^2 ^= 0.95, degrees of freedom (df) = 4 (*P *= 0.92); *I*^2 ^= 0% (Figure 2). The pooled RR for unfavorable outcome at the end of scheduled follow-up was 1.07 (95% CI = 0.93 to 1.22). Heterogeneity: χ^2 ^= 3.10, df = 3 (*P *= 0.38); *I*^2 ^= 3% (Figure 3).

#### Angiographic vasospasm

**Figure 4:**
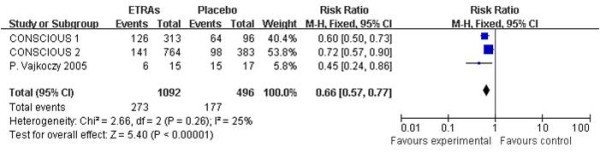
**Risk ratio for angiographic vasospasm**. Comparison between endothelin-receptor antagonists (ETRAs) and placebo in patients with aneurysmal subarachnoid hemorrhage. CI, confidence interval; df, degrees of freedom; M-H, Mantel-Haenszel.

Three studies presented the data for angiographic vasospasm, involving 1,588 patients. The pooled RR for angiographic vasospasm was 0.66 (95% CI = 0.57 to 0.77). Heterogeneity: χ^2 ^= 2.66, df = 2 (*P *= 0.26); *I*^2 ^= 25% (Figure 4). One should note that not all patients in the CONSCIOUS-3 study underwent angiograms (several patients underwent computed tomography angiography instead of digital subtraction angiography) [26], so the data from the CONSCIOUS-3 study were not useful to calculate the rate of vasospasm in this meta-analysis.

#### Delayed cerebral infarction and delayed ischemic neurological deficit

**Figure 5:**
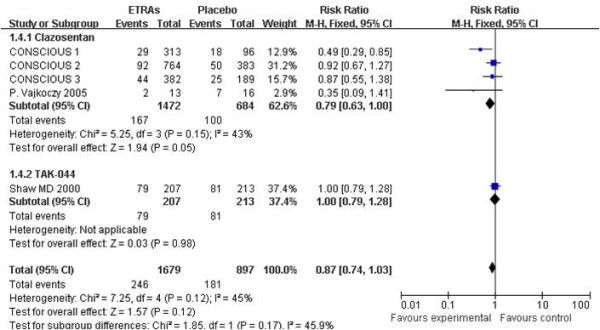
**Risk ratio for delayed cerebral infarctions**. Comparison between endothelin-receptor antagonists (ETRAs) and placebo in patients with aneurysmal subarachnoid hemorrhage and subgroup analysis according to type of ETRA. CI, confidence interval; df, degrees of freedom; M-H, Mantel-Haenszel.

**Figure 6:**
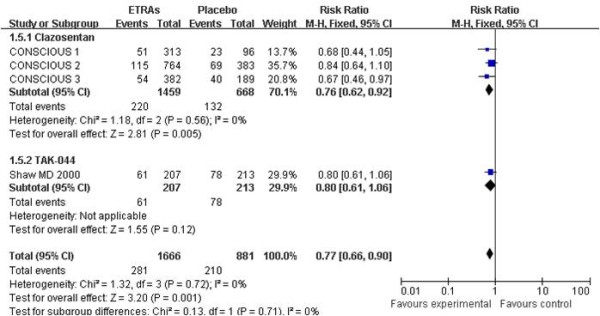
**Risk ratio for delayed ischemic neurological deficit**. Comparison between endothelin-receptor antagonists (ETRAs) and placebo in patients with aneurysmal subarachnoid hemorrhage and subgroup analysis according to type of ETRA. CI, confidence interval; df, degrees of freedom; M-H, Mantel-Haenszel.

All five studies recorded DCI, involving 2,576 patients. The pooled RR for DCI was 0.87 (95% CI = 0.74 to 1.03). Heterogeneity: χ^2 ^= 7.25, df = 4 (*P *= 0.12); *I*^2 ^= 45% (Figure 5). Four studies of 2,547 patients reported DIND. The pooled RR for DIND was 0.77 (95% CI = 0.66 to 0.90). Heterogeneity: χ^2 ^= 1.32, df = 3 (*P *= 0.72); *I*^2 ^= 0% (Figure 6).

#### Adverse events

**Figure 7:**
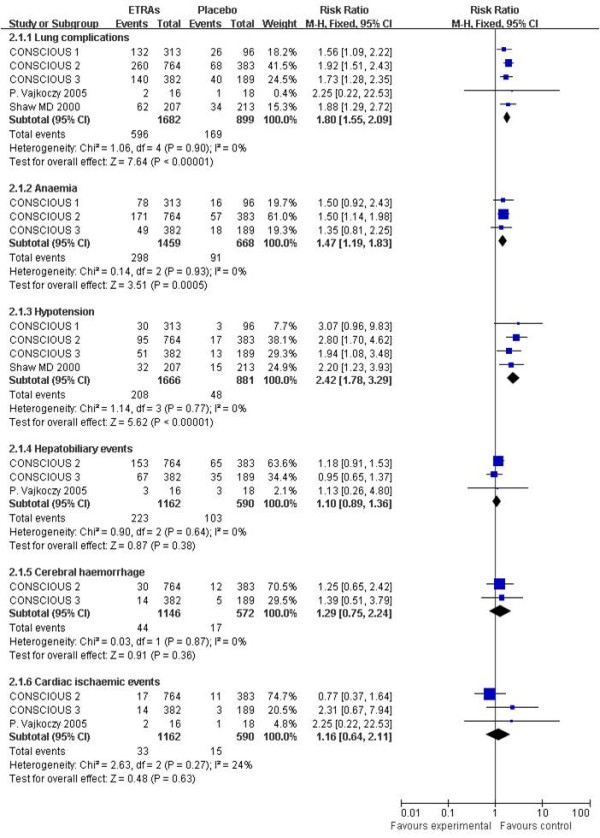
**Risk ratio for adverse events**. Comparison between endothelin-receptor antagonists (ETRAs) and placebo in patients with aneurysmal subarachnoid hemorrhage. CI, confidence interval; df, degrees of freedom; M-H, Mantel-Haenszel.

All five studies reported adverse events. The targeted adverse events under observation mainly contained lung complications, anemia, hypotension, hepatobiliary events, cerebral hemorrhage and cardiac ischemic events. The pooled RR for each adverse event is presented in Figure 7. There were significant increases in lung complications (RR = 1.80, 95% CI = 1.55 to 2.09), hypotension (RR = 2.42, 95% CI = 1.78 to 3.29) and anemia (RR = 1.47, 95% CI = 1.19 to 1.83).

#### Subgroup analysis

**Figure 8:**
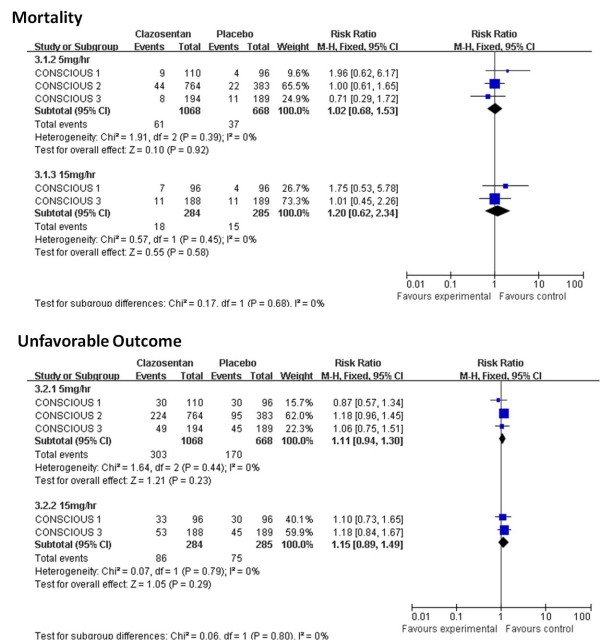
**Mortality and unfavorable outcome according to clazosentan dose**. Subgroup analysis for mortality and unfavorable outcome at the end of follow-up according to the dose of clazosentan. CI, confidence interval; df, degrees of freedom M-H, Mantel-Haenszel.

As the data for subgroup analysis between the coiling group and the clipping group were inadequate and unavailable, we only performed subgroup analysis according to the type (clazosentan vs. TAK-044; Figures 2, 3, 5, 6) and dose (clazosentan, 5 mg/hour vs. 15 mg/hour; Figure 8) of ETRA. The estimate for mortality in the clazosentan group (RR = 1.04, 95% CI = 0.72 to 1.51, *n *= 2,175) was not statistically different from that for the TAK-044 group (RR = 1.00, 95% CI = 0.64 to 1.56, *n *= 420) with *P *= 0.88. There was no difference in treatment effect on unfavorable outcome, DCI and DIND in subgroup analysis according to the type of ETRA. There was also no statistical difference for mortality and unfavorable outcome when analyzed according to the dose of ETRA.

## Discussion

In accordance with the previous meta-analysis, the results of our updated meta-analysis did not find evidence that ETRAs could provide beneficial clinical outcome in patients with SAH. Although ETRAs can significantly reduce angiographic vasospasm (*P *<0.00001) and DIND (*P *= 0.001), they did not improve overall mortality and unfavorable outcome. There was also no significant reduction in DCI. In addition, pulmonary complications, anemia and hypotension were more common in patients receiving ETRAs compared with placebo. The disappointing results do not support the routine use of ETRAs in SAH patients. As hypotension and pulmonary complications are independently associated with poor outcome after SAH [27], further clinical trials of ETRAs in SAH patients should be more carefully formulated and designed.

The present data revealed that there was obvious dissociation between angiographic vasospasm and clinical outcome measurements. This dissociation could result from increased adverse events, methodological problems, insensitivity of clinical outcome, sample size and other possible processes other than angiographic vasospasm contributing to poor outcome [28,29]. Since signs of cerebral ischemia and vasospasm are sometimes reversible, data on vasospasm or DIND in SAH clinical trials may not have been important. In concordance with a recent consensus paper, this meta-analysis also supports the suggestion that DCI on CT scan is a better outcome measure in observational studies and clinical trials than vasospasm and DIND [30]. However, because identifying DCI from all causes of CT hypointensities may be difficult with only a plain CT scan, future advanced techniques and surrogate outcomes should also be designed and introduced [31].

In any meta-analysis, the possibility of publication bias should be considered a potential threat to validity. In this paper, however, we believe that the risk of publication bias affecting the results is minimal due to our extensive and sensitive searching. The high quality of the included RCTs also reduced potential sources of risk of bias. There is no evidence for statistical heterogeneities in pooled analysis of each outcome. However, in this meta-analysis, four limitations should be noted. First, angiography was not routinely performed in all included studies - this would decrease the correlations among vasospasm, DCI, and clinical outcome. Second, explicit definitions for hypotension or pulmonary complications were not classified in the included studies. Similarly, the definition of DIND is not identical between the CONSCIOUS studies and the TAK-044 trial. Third, four included RCTs and our paper use angiographic vasospasm as an outcome measure, but vasospasm of microvessels cannot be visualized on angiography and there is no established criterion for the definition of angiographic vasospasm. Fourth, the treatment of intracranial aneurysm in each included study is not unanimously identical (Table 1). We intended to obtain unpublished data for subgroup analysis (coiling vs. clipping), but no additional data are available to construct the subgroup analysis. The confounding factor of different treatments for intracranial aneurysm should be considered a source for risk of bias and heterogeneities for the pooled data.

## Conclusion

There is no evidence that ETRAs could benefit clinical outcome in patients with SAH. Owing to the increased adverse events, further clinical trials of ETRAs in SAH patients should be more carefully formulated and designed. The present results also suggest that DCI may be a better outcome measure than vasospasm and DIND in clinical trials and observational studies.

## Key messages

• Pilot studies suggested that ETRAs prevent both radiographic vasospasm and DINDs after SAH, but did not indicate that they could improve clinical outcome. Owing to recently published phase 3 clinical trials, the previous meta-analysis on the use of ETRAs to treat aneurysmal SAH has become outdated.

• An up-to-date systemic review and meta-analysis indicates that ETRAs associated with increased adverse events and did not benefit clinical outcome in patients with SAH. Further clinical trials of ETRAs in SAH patients should be more carefully formulated and designed.

## Abbreviations

CI: confidence interval; CT: computed tomography; DCI: delayed cerebral infarction; df: degrees of freedom; DIND: delayed ischemic neurological deficit; ETRA: endothelin-receptor antagonist; RCT: randomized controlled trial; RR: risk ratio; SAH: subarachnoid hemorrhage.

## Competing interests

The authors declare that they have no competing interests.

## Authors' contributions

All authors contributed to the design of the study. JM, HL and SH undertook the searches and screened the citations for eligibility. JM, HL and SH assessed the quality of papers, extracted data, and entered data into RevMan. JM, LM and YL drafted the manuscript. CY moderated disagreements during data collection, analyzed and interpreted data, and helped to draft the manuscript. All authors revised the manuscript and approved the final version.

## Supplementary Material

Additional file 1 a table presenting the PRISMA guidelines. Click here for file

Additional file 2 a diagram presenting the search strategy. Click here for file
