# Supplementary material for: Endothelin-receptor antagonists for aneurysmal subarachnoid hemorrhage: an updated meta-analysis of randomized controlled trials
Source: Crit Care. 2012 Oct 18;16(5):R198. doi: 10.1186/cc11686 (PMC3682300; doi:10.1186/cc11686)
Supplement: Additional file 2 — a diagram presenting the search strategy. [file cc11686-S2.DOCX]

**Search Strategy**

**Database: Ovid MEDLINE (1980 to July 2012) and CENTRAL (*The Cochrane Library*, Issue 2 2012)**

--------------------------------------------------------------------------------

1. exp subarachnoid hemorrhage/ **12642 results**

2 . vasospasm, intracranial/ **2028 results**
3. intracranial aneurysm/ **15554 results**

4. ((subarachnoid or arachnoid) adj6 (haemorrhage$ or hemorrhage$ or bleed$ or blood$)).tw. **14519 results**

5. aneurysm/or aneurysm, ruptured/ **14369 results**

6. ((cerebral or intracranial or cerebrovascular) adj6 (vasospasm or spasm)).tw. **3338 results**

7. SAH.tw. **5707 results**

8. 1 or 2 or 3 or 4 or 5 or 6 or 7 **40378 results**

9. Receptors, Endothelin/ or Receptor, Endothelin A/ or Receptor, Endothelin B/ **7290 results**
10. endothelins/ or endothelin-1/ or endothelin-2/ or endothelin-3/ **18513 results**
11. ((endothel$ or ET-1 or ET$) adj5 antag$).tw. **9656 results**
12. (atrasentan or ambrisentan or Letairis or Volibris or bosentan or bozentan or Tracleer or clazosentan or darusentan or edonentan or enrasentan or sitaxsentan or Thelis or "TAK 044" or tezosentan or Valetri or Veletri).tw. **2313 results**
13. 9 or 10 or 11 or 12 **25924 results**
14. 8 and 13 **365 results**

**Database: EMBASE.com (1980 to July 2012)**

**--------------------------------------------------------------------------------**

#1. 'subarachnoid hemorrhage'/exp OR 'subarachnoid hemorrhage' OR 'subarachnoid haemorrhage'/exp OR 'subarachnoid haemorrhage' AND [1980-2013]/py

**25373 results**

#2. 'intracranial vasospasm'/exp OR 'intracranial vasospasm' OR 'intracerebral vasospasm' OR 'cerebrovascular spasm' AND [1980-2013]/py **4590 results**

#3. 'intracranial aneurysm'/exp OR 'intracranial aneurysm' AND [1980-2013]/py **19596 results**

#4. cerebra* AND ('vasospasm'/exp OR vasospasm) AND [1980-2013]/py **6292 results**

#5. 'SAH' AND [1980-2013]/py **8518 results**

#6. #1 or #2 or #3 or #4 or #5 **41360 results**

#7. endothelin* AND [1980-2013]/py **38846 results**

#8. 'endothelin' NEXT/4 'receptor' AND [1980-2013]/py **35348 results**

#9. 'endothelin' NEXT/5 'antagonist' AND [1980-2013]/py **12017 results**

#10. 'atrasentan'/exp OR 'atrasentan' OR 'ambrisentan'/exp OR 'ambrisentan' OR 'letairis'/exp OR 'letairis' OR 'volibris'/exp OR 'volibris' OR 'bosentan'/exp OR 'bosentan' OR 'bozentan' OR 'tracleer'/exp OR 'tracleer' OR 'clazosentan'/exp OR 'clazosentan' OR 'darusentan'/exp OR 'darusentan' OR 'edonentan'/exp OR 'edonentan' OR 'enrasentan'/exp OR 'enrasentan' OR 'sitaxsentan'/exp OR 'sitaxsentan' OR 'thelis' OR 'tak 044'/exp OR 'tak 044' OR 'tezosentan'/exp OR 'tezosentan' OR 'valetri'/exp OR 'valetri' OR 'veletri'/exp OR 'veletri' AND [1980-2013]/py **6957 results**

#11. #7 or #8 or #9 or #10 **38913 results**

#12. #11 and #6 **698 results**

#13. #12 AND [humans]/lim AND [1980-2013]/py **386 results**

**Database: Pubmed (1980 to July 2012)**

**--------------------------------------------------------------------------------**

#1. subarachnoid hemorrhage or subarachnoid haemorrhage[Title/Abstract] **20686 results**

#2. intracranial vasospasm OR intracerebral vasospasm OR cerebrovascular spasm[Title/Abstract]  **3746 results**

#3. intracranial aneurysm[Title/Abstract] **2322 results**

#4. SAH[Title/Abstract] **6022 results**

#5. #4 OR #3 OR #2 OR #1  **24012 results**

#6. endothelin*[Title/Abstract] **23481 results**

#7. Receptors, Endothelin/ or Receptor, Endothelin A/ or Receptor, Endothelin B/[Title/Abstract] **9484 results**

#8. antag*, Endothelin*/[Title/Abstract]  **9813 results**
#9. #6 OR #7 OR #8 **24218 results**

#10. #9 and #5  **351 results**

**Database: Science Citation Index Expanded (SCI-EXPANDED) (from 1980 to May 2012)**

#1. Topic=(subarachnoid hemorrhage or subarachnoid haemorrhage) [**Approximately 34,488**](http://apps.webofknowledge.com/summary.do?product=UA&doc=1&qid=3&SID=Z2HiIB@GKhiJclpEm65&search_mode=GeneralSearch) **results**

#2. Topic=(intracranial vasospasm OR intracerebral vasospasm OR cerebrovascular spasm) [**Approximately 5,648**](http://apps.webofknowledge.com/summary.do?product=UA&doc=1&qid=4&SID=Z2HiIB@GKhiJclpEm65&search_mode=GeneralSearch) **results**

#3. Topic=(intracranial aneurysm) [**Approximately 28,472**](http://apps.webofknowledge.com/summary.do?product=UA&doc=1&qid=5&SID=Z2HiIB@GKhiJclpEm65&search_mode=GeneralSearch) **results**

#4. Topic=(SAH) [**Approximately 11,330**](http://apps.webofknowledge.com/summary.do?product=UA&doc=1&qid=6&SID=Z2HiIB@GKhiJclpEm65&search_mode=GeneralSearch) **results**

#5. #4 OR #3 OR #2 OR #1 **[Approximately 56,026](http://apps.webofknowledge.com/summary.do?product=UA&doc=1&qid=7&SID=Z2HiIB@GKhiJclpEm65&search_mode=CombineSearches) results**

#6. Topic=(Receptors, Endothelin/ or Receptor, Endothelin A/ or Receptor, Endothelin B/) [**Approximately 25,910**](http://apps.webofknowledge.com/summary.do?product=UA&doc=1&qid=9&SID=Z2HiIB@GKhiJclpEm65&search_mode=GeneralSearch) **results**

#7. Topic=(antag*, Endothelin*) [**Approximately 18,538**](http://apps.webofknowledge.com/summary.do?product=UA&doc=1&qid=10&SID=Z2HiIB@GKhiJclpEm65&search_mode=GeneralSearch) **results**

#8. Topic=(atrasentan or ambrisentan or Letairis or Volibris or bosentan or bozentan or Tracleer

or clazosentan or darusentan or edonentan or enrasentan or sitaxsentan or Thelis or "TAK 044" or

tezosentan or Valetri or Veletri) [**Approximately 5,734**](http://apps.webofknowledge.com/summary.do?product=UA&doc=1&qid=11&SID=Z2HiIB@GKhiJclpEm65&search_mode=GeneralSearch) **results**

#9. #8 OR #7 OR #6 **[Approximately 31,143](http://apps.webofknowledge.com/summary.do?product=UA&doc=1&qid=14&SID=Z2HiIB@GKhiJclpEm65&search_mode=CombineSearches) results**

#10. #9 AND #5 [**571**](http://apps.webofknowledge.com/summary.do?product=UA&doc=1&qid=15&SID=Z2HiIB@GKhiJclpEm65&search_mode=CombineSearches) **results**
